# High-Risk Human Papillomavirus (HR-HPV) DNA Detection in Mouthwashes for Diagnosis of HPV-Driven Oropharynx Cancer and Its Curative Therapy—A Feasibility Study

**DOI:** 10.3390/jcm11195509

**Published:** 2022-09-20

**Authors:** Gera Loermann, Marlen Kolb, Dusan Prascevic, Julia Siemert, Susanne Wiegand, Veit Zebralla, Markus Pirlich, Matthäus Stöhr, Andreas Dietz, Theresa Wald, Gunnar Wichmann

**Affiliations:** 1 Clinic for Otorhinolaryngology, Head and Neck Surgery, University Hospital Leipzig, Liebigstr. 10-14, 04103 Leipzig, Germany; 2 Peter Debye Institute for Soft Matter Physics, Leipzig University, Linnéstraße 5, 04103 Leipzig, Germany

**Keywords:** head and neck squamous cell carcinoma (HNSCC), oropharyngeal squamous cell carcinoma (OPSCC), human papillomavirus (HPV), HPV-related cancer, p16 expression, mouth rinse, genotyping

## Abstract

Detection of p16 through immunohistochemistry (IHC) is the standard for determining the HPV status of the tumor according the TNM eighth edition released in 2017 and has become crucial for determining the HPV status of oropharyngeal squamous cell carcinomas (OPSCC) with direct impact on staging and prognostication. In recent years, detection of HPV DNA in mouthwashes has been proposed as a noninvasive alternative, both for OPSCCs and for other head and neck squamous cell carcinomas (HNSCCs). However, the prospect of using the mouthwashes to monitor the response to therapy is unclear. To evaluate the effect of curative therapy on the detection of HPV DNA, we performed a prospective study comparing the detection frequency of high-risk HPV DNA (HR-HPV-DNA) in pre- and post-therapy mouthwashes. We collected 137 mouthwashes from 88 pathologically confirmed HNSCC patients for DNA isolation and HPV genotyping with the Inno-LiPA assay. We show that HPV DNA in pretherapeutic mouthwashes can detect HPV-driven HNSCCs with a sensitivity of 50.0% and specificity of 85.4%, alongside a high negative predictive value of 79.5% and an accuracy of 74.5%. Furthermore, we observed a notable decrease in the detection frequency of HR-HPV-DNA after successful treatment (pre-therapy 50.0% (9/18) versus post-therapy 9.7% (3/28)). However, the comparatively low sensitivity regarding detection of HPV-driven OPSCC argues against its use in clinical routine.

## 1. Introduction

The significance of human papillomavirus (HPV) in head and neck squamous cell carcinoma (HNSCC), particularly in oropharyngeal squamous cell carcinomas (OPSCC), has been extensively researched in recent years. Along with smoking and alcohol, infection with oncogenic HPV subtypes followed by neoplastic transformation drives the development of OPSCC. Therefore, HPV is considered as one of the main risk factors for OPSCC, and the number of newly diagnosed OPSCC cases with HPV-associated OPSCC is clearly increasing [1]. Patients with HPV-related OPSCC, in contrast to patients with HPV-negative OPSCC, however, have a significantly more favorable prognosis despite the presence of loco-regional metastasis [2,3].

According to several studies, HPV-driven tumors (detectable HPV DNA and HPV E6*I mRNA) were shown to be genetically diverse and are now considered as a special subgroup compared to HPV-negative OPSCC [4,5,6,7,8,9]. During HPV-driven carcinogenesis, viral proteins, particularly the two HPV early proteins E6 and E7, interact with cell-cycle proteins causing neoplastic transformation. In particular, E6 leads to ubiquitinylation and proteolytic degradation of p53, whereas E7 of oncogenic HPV subtypes interacts with histone demethylases triggering loss of trimethylation of histone-lysine 27, resulting in a secondary strong expression of the tumor suppressor protein p16^INK4A^ (p16). As direct detection of E6 and E7 is challenging, the analyses of mRNA encoding these proteins, e.g., E6*I, a transcript spanning E6 and E7, is often used to define active involvement of HPV in neoplastic transformation driving the disease [4,8]. Consequently, an alternative to laborious detection of HPV subtype-specific E6*I mRNA is the use of the surrogate marker p16^INK4A^ followed by detection of HPV DNA [10,11,12]. However, the latter is often not performed as HPV DNA and/or RNA is detected in most p16^+^ OPSCCs [11,13]. However, the sensitivity and especially the specificity are decreased by about 6% and 17%, respectively [11]. Despite the reduced specificity caused by omission of HPV DNA and/or RNA analyses, the current eighth edition of the TNM classification (TNM 2017) focuses on p16 immunohistochemistry (IHC) and provides a different definition of N categories and a general downstaging of p16^+^ OPSCC based on the significantly superior prognosis of HPV-related OPSCC [14,15]. In clinical practice, and as recommended by TNM 2017, the HPV status in HNSCC/OPSCC patients is mostly determined by p16 IHC as a surrogate marker [14]. However, this approach is currently under discussion, as some studies have proven that 15–25% of OPSCCs with p16 detection are in fact HPV negative [16,17,18,19,20,21,22]. This can be due to mutated CDKN2A, the gene encoding p16, or flawed p16^+^ IHC by counting p16^+^ senescent cells without proliferation, a substantial pitfall if p16 is stained solely [10]; the latter problem could be solved by IHC staining for Ki-67 expression [10]. Moreover, p16 IHC requires formalin-fixed paraffin-embedded (FFPE) tissue and, therefore, cannot be used for monitoring and screening purposes. The ongoing debate emphasizes the need for new markers that are able to facilitate reliable diagnosis of HPV-related OPSCC and their follow-up in clinical routine. Monitoring of antibodies to HPV early proteins in serum or plasma may overcome this issue and is of high sensitivity and specificity [23]. The instability of reagents requiring fresh preparation of antigens limits transferability of the method into clinical routine. Moreover, this method requires at least drawing of blood and is, therefore, an invasive diagnostic method. Therefore, some authors examined mouthwash solutions as possible alternatives for sampling of HPV DNA.

Using 10 mL of phosphate-buffered saline, Koslabova and colleagues were able to detect HPV DNA in pre-therapy mouth rinses of 53.2% (75/141) HNSCC patients [24]. They reported that, of 83 patients with high-risk HPV subtype (HR-HPV) DNA-positive tumor tissue, 64 (77.1%) had HR-HPV detected in pre-therapy oral rinses with a concordant HPV subtype present in 62 of these (96.9%). Of 58 patients with HPV-negative tumors, only 15.5% (9/58) had HR-HPV DNA detected in their oral lavage fluid. A report from Australia suggested high sensitivity (92.9%) and 100% positive predictive value of HPV16 DNA detection in saliva as 39 of their 42 p16-positive HNSCC had a positive end-point PCR [25]. They obtained their samples via gargling for 1 min with 10 mL of 0.9% saline [25]. Wang and collaborators reported that the presence of HPV DNA in bodily fluids and mouth rinses in particular represents a very convenient marker for HPV-related OPSCC [26]. They reported no differences when using one of two protocols: either utilizing sampling of saliva from patients who were asked to allow saliva to collect in the floor of the mouth for 5 min without swallowing before spitting into the collection vial or swishing 15 to 20 mL of 0.9% sodium chloride in their mouths for 10 to 15 s before spitting into the collection tube [26]. This suggested that an oral rinse independent from carrier fluids used was sufficient for obtaining material for HPV detection and genotyping. However, DNA purification and preparation for detection via polymerase chain reaction (PCR) may have an impact on the sensitivity and specificity of HPV detection [27,28].

As demonstrated by Rosenthal and colleagues, the use of mouthwash could be a highly specific test for diagnosis of oropharyngeal cancer [29]. Unfortunately, they did not report about the potential use of HPV detection in mouth rinses to assess therapeutic response in terms of therapy-related reduction in the amount of detectable HPV DNA or even a complete loss of detectable HPV DNA after curative treatment. According to the hypothesis that successful eradication of HPV-driven OPSCC potentially results in loss of HPV DNA in saliva, we focused on the detection of high-risk HPV DNA (HR-HPV-DNA) in mouthwashes taken before and after treatment. To this end, we designed a prospective feasibility study to assess the following: (i) the presence of HPV DNA in pre-therapy mouthwashes; (ii) the presence of HPV DNA in post-therapy mouthwashes; (iii) the correlation of HPV in pre- and post-therapy mouthwashes with the HPV status of OPSCC and HNSCC of other localization; (iv) the capability of HPV DNA in post-therapy mouthwashes to predict the patient outcome.

## 2. Experimental Section

### 2.1. Subjects and Materials

The study was approved by the Ethics committee of the Medical Faculty of the University Leipzig (votes 201-10-12072010, 202-10-12072010, and 341-15-ff; 28.10.2015) according to the Helsinki Declaration II. Eligible were patients with histologically confirmed HNSCC treated in the ENT department of the University Hospital Leipzig. The treatment of HNSCC followed the recommendations according to the personalized decisions of the multidisciplinary head and neck tumor conference of the University Hospital Leipzig based on NCCN guidelines. According to sample size estimation by taking into account the expected frequency of HPV-driven HNSCC [8] and allowing a maximum error of ε = 2.5%, we expected a minimal number of ≥31 HR-HPV DNA^+^ cases being required to answer the question about significance in the correlation of HR-HPV DNA detection in mouthwashes and HPV-driven HNSCC. After obtaining their informed consent, we accrued 88 HNSCC patients.

### 2.2. Collection of Mouth Rinses

To investigate HPV DNA present in their throat and oral cavity, patients rinsed their mouth with 20 mL of sterile-filtrated tap water for 1 min by switching after 10 s each from gargling to purging. The fluid was sampled in a tube and immediately transferred at room temperature into the lab. Mucosa cells were then pelleted by 10 min centrifugation at 4000× *g* and stored up to a maximum of 3 months at −80 °C until DNA was isolated. DNA was extracted from the pellet using QIAamp DNA Blood Mini Kit on a QIAcube according to the manufacturer’s instructions (QIAGEN, Hilden, Germany). Before storage at −20 °C, yield and purity of DNA were measured on a Synergy 2 (BioTek instruments, Bad Friedrichshall, Germany) using a Take 3 plate (BioTek) for measurement of absorbance at λ_1_ = 260 nm and λ_2_ = 280 nm and AE buffer (the extraction buffer from the QIAamp DNA Blood Mini Kit) for reference. Preliminary to HPV genotyping, we quantified the double-strand DNA (dsDNA) content of samples using a Qubit™ 4 Fluorimeter (Thermo Fisher Scientific, Singapore) applying the highly sensitive dsDNA kit.

### 2.3. HPV Genotyping

For HPV detection and genotyping, the line probe assay Inno-LiPa HPV Genotyping Extra II (CE) kit (Innogenetics, Gent, Belgium) was used [8]. Based on the reverse hybridization principle, this assay allows for the identification of 28 different HPV genotypes. According to the kit manufacturer’s instructions, an end-point PCR utilizing the SPF10 degenerated primers and 100 ng of dsDNA was performed on a DNA Engine (Bio-Rad, Munich, Germany). This was followed by detection of specific HPV sequences in a 65 bp region of the L1 region of the HPV genome; positive controls for sufficient input of genomic DNA (amplification/hybridization of a HLA-DPB fragment) and the staining reaction are included and visualized by dedicated bands. We processed the hybridization of these amplicons to reverse HPV type-specific DNA probes spotted on stripes on an Auto-LiPA (Innogenetics) utilizing the protocol provided and programmed by Innogenetics. The presence of certain genotypes is visualized by particular patterns of stained bands which were detected and interpreted by means of an Epson scanner (Epson Perfection 4490 Photo; Epson, Suwa, Japan) utilizing the software provided by Innogenetics (LiRAS™ for LiPA HPV v. 2.00). Using the HPV18-positive cell line KB [30,31,32] to assess the lower limit of detection of HPV18 in 100 ng of genomic DNA (the amount utilized in the initial PCR step), we found full concordant staining in triplicate measurements down to 100 HPV18-positive KB cells among 1 × 10^6^ peripheral blood mononuclear cells (PBMCs) spiked into 20 mL of sterile filtered tap water. This finding corresponds to reliable detection of 0.1 ng of KB-derived DNA in a background of 100 ng of DNA (~8 cells containing HPV18 among >8000 cells).

### 2.4. Classification of HNSCC as HPV-Driven

HNSCC were classified on the basis of the results of HPV genotyping into HR-HPV-DNA^+^ (presence of DNA of any HR-HPV subtype related to high or intermediate risk; HR-HPV-DNA^+^) or HPV-DNA^−^. As DNA of HPV16 was the predominant HPV subtype in HR-HPV-DNA^+^ HNSCC, RNA samples of HNSCC positive for this subtype separately underwent the E6*I mRNA RT-PCR assay for HPV16 detection, as previously described [4,7,23]. We defined a sample as HPV16 RNA^+^ whenever HPV16 E6*I transcripts were detected. Tumors were classified on the basis of combined results from HPV-DNA genotyping and detection of either HPV16 DNA^+^ RNA^+^ or HR-HPV DNA^+^ plus p16 positivity of >70% of tumor cells in immunohistochemistry by applying the CINtec^®^ plus kit (Roche, Mannheim, Germany) as HPV-driven (HPV16 DNA^+^ RNA^+^ or HR-HPV DNA^+^ p16^+^) or not [7,20].

### 2.5. Statistical Analysis

The statistical analyses were performed using SPSS version 27 (IBM Corporation, Armonk, New York, NY, USA) and included *Pearson’s* chi-square (*χ*^2^) tests, *Fisher’s* exact tests, and *McNemar* tests to assess differences between categorical variables.

## 3. Results

Until achieving the predefined inclusion limit of 32 p16^+^ cases with HR-HPV DNA in their primary HNSCC, 88 patients were included (Table 1). From these 88 cases in total, 59 (67.0%) pre- and 78 (88.6%) post-therapy mouthwash pellets, respectively, had sufficient amount of DNA for HPV analyses. The time interval between pre- and post-therapy sampling was 7.5 (95% CI 6.2–8.7) months. The comparison of HR-HPV DNA detection in pre-therapeutic mouthwashes and HNSCC tumors was possible in 31 cases with OPSCC (52.5%) and 28 cases with primary in other localizations (47.5%).

Of 21 HR-HPV DNA-negative OPSCC patients, in 16 cases (76.2%), the HPV status of their tumor was not HPV-related (p16-), whereas five OPSCC patients (23.8%) had HPV-driven tumors, but their mouthwashes were not found to be positive for HPV despite each having a sufficient PCR control. HR-HPV DNA was detected in mouth rinses of 7/12 (58.3%) OPSCC patients with HPV-driven tumors (sensitivity 58.3%, 50% including other HNSCC localizations). However, HR-HPV DNA was additionally detected in 3/19 OPSCC (15.8%) with p16-negativity of the primary lesion (specificity 84.2% in OPSCC, 85.4% including HPV-driven HNSCC; Table 2). The sensitivity of HPV16 detection in pre-therapeutic mouthwashes from OPSCC patients with HPV-driven tumors was 25%, and the specificity was 94.7% (Table 3). Including other localizations of the primary, HPV16 detection was 27.8% sensitive and 95.1% specific (Table 3).

Overall, there was a significant correlation of HR-HPV DNA detection in pre-therapeutic mouth rinses and HPV-driven OPSCC (*r* = 0.443; *p* = 0.014), leading to an accuracy of the findings in 74.2% of mouthwashes regarding diagnosis of HPV-driven OPSCC. When it comes to detection of other HPV-driven HNSCC with primary localizations outside the oropharynx (ICD-10-C02, C04, C12, C13, C32; Table 2), the accuracy was comparable (75.0%), but the sensitivity was only 33.3%, making the identification of HPV-related disease exceedingly difficult (χ^2^ = 1.247, *p* = 0.264; *p* = 0.606 after continuity correction for *n* < 30). Summarizing all localizations, the identification of HR-HPV DNA in pre-therapy mouth rinses correlated significantly with HPV-driven HNSCC (*p* = 0.004, continuity-corrected *p* = 0.011; Fischer’s exact test *p* = 0.008; accuracy 63.8%). We found a specificity of 85.4% for HPV-driven HNSCC whenever HR-HPV DNA was detected in pre-therapy mouth rinses. However, if the correlation between HPV16 in the pre-therapeutic mouth rinse and the probability to identify an HPV16-driven tumor is considered, HPV16 detection was possible in only 27.8% of cases. This indicates a sensitivity problem. Additionally, we were unable to detect HR-HPV DNA in the majority of pre-therapy mouth rinses (50% of HPV-driven tumors had no HR-HPV DNA in their mouthwash). In summary, we demonstrated the limited ability to detect HPV-driven tumors by finding reasonable specificity of 85.4%, accompanied by a high negative predictive value of 79.5% and an accuracy of 74.6%, but a low sensitivity of 50%.

The comparison of HR-HPV DNA detection in post-therapeutic mouth rinses and HNSCC tumors was possible in 78 cases, 49 cases with OPSCC (62.8%) and 29 cases with primary in other localizations (37.2%). In 49 post-therapy mouthwashes from OPSCC patients, only two (8%) post-therapy mouthwashes contained HR-HPV DNA, and both were from patients without HPV-driven primaries. Independent from treatment modalities applied, the 24 cases with HPV-driven OPSCC among OPSCC were free from detectable HR-HPV DNA (100% negative) after curative treatment. Despite low sensitivity regarding identification of HPV-driven HNSCC (as detected in the pre-therapy mouthwashes), the post-therapy HR-HPV negative mouthwashes from prior (pre-therapy) HR-HPV DNA^+^ mouthwashes from HPV-driven OPSCC patients could be associated with their status “cured” (no sign of disease) at time of post-therapy sampling. This is in line with the findings above described of the comparison between pre-therapy mouthwashes and the tumors’ HPV status. In these, absence of HR-HPV had a high negative predictive value of 79.5% and an accuracy of 63.8% in identification of HPV-driven disease.

The detection of other HPV types in post-therapeutic mouthwashes in a further eight (three HPV-driven and five not HPV-driven) out of 49 (16.3%, 12.5%/20%) OPSCC patients demonstrated a lack in correlation of HR-HPV DNA in post-therapeutic specimens with the initial HPV status.

Moreover, the only detection of HR-HPV in post-therapeutic mouthwashes of patients with a primary lesion outside the oropharynx 1/29 (3.4%) was in a case with a non-HPV-driven tumor. This, together with the lack of any correlation of HR-HPV detection in post-therapy mouthwashes with the HPV status of the primary tumor (HPV-driven or not) suggests a lack of correlation of HR-HPV DNA in post-therapeutic specimens with the initial HPV status. Absence of HR-HPV DNA in post-therapy mouthwashes, however, indicates successful treatment if there was detection of HR-HPV DNA in pre-therapy samples from HPV-driven OPSCC according to our finding in the small subgroup of HPV-driven OPSCC (Table 4).

## 4. Discussion

Investigating the hypothesis that eradication of HPV-driven OPSCC will lead to loss of detectable HR-HPV in saliva, we demonstrated with a small but sufficiently large sample of 88 HNSCC patients including 49 cases with OPSCC (62.8%) that detection of HR-HPV-DNA in mouth-rinsing solutions before and absence of HR-HPV-DNA after treatment correlates with successful eradication of the HPV-driven OPSCC. However, pre-therapy mouthwashes do not contain in every HPV-driven HNSCC a sufficient amount of HR-HPV DNA and, therefore, do not allow for reliable detection of HPV-driven disease.

We selected the patients for our study by consecutively inviting all patients with histologically confirmed HNSCC to participate in the study. According to prospective sample size estimation, we expected a minimal number of ≥31 HR-HPV DNA^+^ cases being required to answer the question about a potential correlation of HR-HPV DNA detection in mouth rinses and HPV-driven HNSCC, with the same case number for post-therapy investigations (without correcting for multiple analyses). In total, 53 (60.2%) out of 88 patients accrued were OPSCC patients, and 35 (39.8%) were patients with another tumor localization in the head and neck region. Of 53 OPSCC, 25 (47%) were HPV-driven (HR-HPV DNA^+^ RNA^+^ or HR-HPV DNA^+^ p16^+^), and 28 (53%) had either a negative p16 or a negative HPV status.

The most common method to detect HPV in OPSCC/HNSCC patients currently used in clinical practice and recommended by the AJCC and UICC TNM Committee is p16 IHC as a surrogate marker for HPV-related disease. This indirect cellular marker points to the upregulation of CDKN2A transcription translated into p16 overexpression triggered by expression of HPV proteins, particularly E7. Therefore, p16^INK4A^ IHC is considered an adequate surrogate marker for neoplastic transformation through HPV infection (20). However, the pooled sensitivity of p16^INK4A^ IHC of 94% (95% confidence interval (CI) 91–97%) and specificity of 83% (CI 78–88%) compared to HPV DNA and RNA detection demonstrates some limitations of sole p16^INK4A^ IHC and is not superior to HPV DNA PCR (sensitivity 98% (CI 94–100%), specificity 84% (CI 74–92%)). The most used threshold to declare HNSCC as p16^+^ is a nuclear and/or cytoplasmic staining of more than 70% of tumor cells [11,33]. However, there is a controversial discussion about the sole use of p16 positivity as the marker for detecting HPV-driven OPSCC. Some authors reported 15–20% of p16^+^ OPSCC being HPV16-negative in the polymerase chain reaction followed by in situ hybridization [16,17,18,19,20,21]. A possible explanation for false-positive findings could be that mutations in the p16 gene CDKN2A or in RB1 may also lead to p16 overexpression [34]. Our own investigations demonstrated about 23% of p16^+^ OPSCC to be not HPV-driven (no detectable HPV DNA and/or RNA) associated with impaired outcome in some p16^+^ OPSCC [22]. Thus, there is a risk for patients with HPV-negative OPSCC inadvertently being placed into a group of patients with better prognosis potentially receiving (inadequately) a de-escalation of treatment intensity [35]. If HPV-driven (p16^+^ HR-HPV DNA^+^) tumors are treated differently in the future, this could have immense detrimental consequences given the outlined circumstances. Therefore, the recommendation is to determine both HR-HPV DNA and transcriptional activity of HR-HPV, the prerequisite for neoplastic transformation. Transcriptional activity can be detected via viral mRNA transcripts of oncogenes E6 and E7 (HPV16 E6*I, specifically) using RT-PCR, which is considered the current gold standard [4,11]. However, detection of HPV16 E6*I transcripts requires high-quality RNA derived from unfixed, deep-frozen tumor samples. This makes the unambiguous identification of tumor cells methodologically challenging and very time-consuming. Another disadvantage in addition to the limited availability of snap-frozen tissue for RNA extraction and the related potential pre-analytic dropout of samples is the high cost of this method requiring individually designed primers for the various HR-HPV subtypes. The current gold standard can be nearly replaced by simultaneous IHC for detection of p16^INK4A^ and Ki-67 [10] or by combining p16^INK4A^ IHC and HR-HPV-DNA PCR (sensitivity 93%, CI 87–97%, specificity 96%, CI 89–100% [11]). Today, there is no reliable screening test for detecting HPV-driven OPSCC for clinical routine.

In the search for a viable screening tool for HPV in OPSCC patients, Rosenthal et al. (2017) previously developed an oral rinse test that could serve as an early detection test for HPV-driven oropharyngeal carcinomas [29]. Earlier reports by Koslabova et al., suggested a sensitivity of 77.1% for detecting HPV-positive HNSCC with a high concordance between HPV subtypes detected in tumor tissue and oral rinses [24]. Moreover, Chai et al., reported concordance above 70% [25].

We wanted to investigate if there are hints that DNA detection in mouthwashes could serve as a screening tool for recurrence in the follow up of HPV-related OPSCC patients. The main advantage of obtaining a mouthwash for HPV detection is the noninvasive nature of the method that easily can be integrated into clinical routine. In addition, family doctors or dentists could regularly check the HPV status of OPSCC patients who have already completed treatment and are under follow-up.

Unfortunately, our analyses of HPV in pre-therapy taken mouthwashes of HNSCC patients demonstrate a relevant sensitivity problem (only 58.3% of HPV-driven OPSCC and 33.3% of other HNSCC could be detected). However, the method itself is sufficiently sensitive for reliable detection and genotyping of HPV in a mouthwash containing eight HPV18-positive cells among 8000 exfoliated cells with full concordant staining. Full concordant staining refers to full representation of staining patterns for typing of the HR-HPV subtype and presence of all internal quality controls. The detection of any HPV DNA might eventually be possible also at lower frequencies. This means that the sensitivity problem relates probably to other so far unknown causes. Loss of the L1 gene used for HPV genotyping or mutations in L1 preventing proper binding of primers and amplification during PCR could be excluded as the same HPV genotyping kit was used for HPV detection and genotyping of tumor tissue. Therefore, shedding only few malignant cells with intact HPV DNA and degradation of such cells or debris containing HPV DNA seem to be hypothetical explanations requiring experimental proof. Despite this sensitivity problem regarding the detection of HR-HPV in HPV-related OPSCC, for cases with pre-therapeutic detectable HR-HPV DNA, the analyses post-treatment mouthwashes appear to be able to indicate the successful cure of HPV-related OPSCC by eradication of HPV DNA (Table 4). In contrast, those with HPV-driven HNSCC and HR-HPV DNA present after about 6 months since completed treatment in curative intent are at high risk for relapse (Table 4). This is in line with findings of Ekanayake Weeramange and coauthors [36]. In saliva samples taken at diagnosis of OPSCC, they detected HR-HPV DNA in 81.4% of p16-positive cases. Prognosis in salivary HR-HPV-positive OPSCC patients was favorable compared with that in salivary HR-HPV DNA-negative patients (event-free survival, hazard ratio 0.42 (95% CI, 0.21–0.81; *p* = 0.010)) [36]. However, in line with our findings of HR-HPV DNA detectable in few patients with not HPV-related HNSCC and pre-therapy negative mouthwashes, even the specificity might be too low. As data from Gillison et al., suggest [37], asymptomatic persons and healthy adults may also have oral HR-HPV infection and HR-HPV DNA detectable in mouth rinses with a prevalence peak among individuals aged 60 to 64 years (11.4%; 95% CI, 8.5–15.1%). As this is a very relevant age group among HNSCC patients, specificity of HR-HPV DNA positivity for detection of HNSCC appears to be limited by this offset. However, Martin-Gomez and colleagues [38] reported results of a prospective study that aimed to detect relevant HPV genotypes in oral gargle samples of men with newly diagnosed OPSCC. They also used a highly sensitive reverse hybridization assay to detect HPV DNA in both saliva samples and available tumor specimens as in this study. Among 204 patients, they found that 175 of the tumor specimens (86%) stained positive for p16, whereas 168 of the oral gargles (83%) were positive for HPV. They detected HPV16 in 143 of 172 tumor specimens (83%) and 128 of 203 oral gargle samples (63%); the agreement for HPV16 in the 171 cases that had paired gargle sample and tumor specimens was 74% [38]. In an invited commentary [39], Ramirez and Zevallos critically discussed that the data from their study [38] showed that HPV DNA from oral gargle samples is an imperfect surrogate biomarker due to the discordance in over one-quarter of patients. Earlier reports with much higher detection rates with sensitivity and specificity as Chai et al. [25] and Kreimer et al. [40] were obtained in other than the routine setting of a typical ear, nose, and throat clinic (like our outpatient clinic). A substantially deviating distribution of patient characteristics, e.g., increased simultaneous infection with the human immunodeficiency virus (HIV) or even inclusion of HIV-related acquired immunodeficiency syndrome (AIDS) patients with CD4^+^ cell counts of <200 cells per milliliter may have led to much higher sensitivity and specificity of HPV detection in oral rinses obtained from these patients, as, in such patient cohorts, the highest detection rate of HPV in oral samples was reported [40].

In 2018, Gipson and his research team published the meta-analysis “Sensitivity and specificity of oral HPV detection for HPV-positive head and neck cancer” [41]. They also concluded that oral HPV detection in HNSCC patients has good specificity but only moderate sensitivity. This led them to the assumption that the benefit of oral HPV detection for screening for HNSCC in healthy populations is likely to be limited as there would be many false negatives and false positives. However, the possibility of considering the analyses as screening for the successful cure of HPV was not discussed. Rettig et al. [42] published a paper comparing pre- and post-therapy mouthwashes. HPV16 DNA in oral rinses was common at diagnosis (67 of 124 participants [54%]). In contrast, after treatment, oral HPV16 DNA was detected in six (5%) patients, including five with HPV16 DNA also detected at diagnosis (persistent oral HPV16 DNA). While observing 2 year disease-free (DFS) and overall survival (OS) of 92% (95% CI, 94–100%) and 98% (95% CI, 93–99%), oral persistent HPV16 DNA correlated with worse DFS (hazard ratio, 29.7 (95% CI, 9.0–98.2)) and OS (hazard ratio, 23.5 (95% CI, 4.7–116.9)). This is in line with our findings (Table 4). However, our study had some limitations that need to be considered. On the one hand, we integrated both OPSCC patients and HNSCC patients, as the number of study participants would have otherwise been too small within the given time. If the study was conducted only with OPSCC patients who are significantly more likely to be affected by HPV-driven tumors, the results would probably have been clearer. We also determined that, in spite of often low DNA yields and resulting low DNA concentrations, HPV detection was successful. The hybridization controls used as internal controls for successful amplification of human control DNA ensured the validity of the analyses and showed that the lack of HPV detection was not due to too low DNA concentrations but probably due to too high background of DNA from healthy cells and potential contaminants including DNA from the oral microbiome and various microorganisms. A nested PCR may potentially allow overcoming the sensitivity problem in some regard. Executing a nested PCR, however, substantially increases complexity and cost for analyses, making such an approach unfeasible for routine screening purposes.

Summarizing our feasibility study’s results, the use of mouthwashes to detect HPV-driven HNSCC via detection and genotyping of HPV cannot be recommended as a diagnostic for HPV-driven HNSCC due to low sensitivity and specificity. As we detected HPV DNA independent from prior detection of HPV DNA in pre-therapeutic mouthwashes samples or not, particularly the pre-therapeutic HPV status of the primary HNSCC in some cured patients, there is also a low reliability regarding discrimination between residual disease and cure. We deem sole HPV DNA detection and genotyping as inadequate for the desired purpose.

## Figures and Tables

**Table 1:** Characteristics of patients included in this study.

Characteristics		Total *n (%)*		HPV-Driven ^1^*n (%)*		Not HPV-Driven ^2^*n (%)*		*p*-Value *
Group Size		88	(100.0)	32	(36.4)	56	(63.6)	
Age (years)	≤50	9	(10.2)	2	(6.3)	7	(12.5)	0.4218
	>50–60	40	(45.5)	13	(40.6)	27	(48.2)	
	>60–70	23	(26.1)	8	(25.0)	15	(26.8)	
	>70–80	12	(13.6)	7	(21.9)	5	(8.9)	
	>80	4	(4.5)	2	(6.3)	2	(3.6)	
Sex	Female	22	(25.0)	7	(21.9)	15	(26.8)	0.6088
	Male	66	(75.0)	25	(78.1)	41	(73.2)	
OPSCC vs. other	OPSCC	53	(60.2)	25	(78.1)	28	(50.0)	**0.0095**
	other	35	(39.8)	7	(21.9)	28	(50.0)	
Smoking	never	23	(26.1)	12	(37.5)	11	(19.6)	**0.0131**
	former	31	(35.2)	14	(43.8)	17	(30.4)	
	current	34	(38.6)	6	(18.8)	28	(50.0)	
Smoking categories	non-smoker	23	(26.1)	12	(37.5)	11	(19.6)	0.0980
	≤10 PY	4	(4.5)	1	(3.1)	3	(5.4)	
	11–30 PY	30	(34.1)	13	(40.6)	17	(30.4)	
	31–60 PY	25	(28.4)	4	(12.5)	21	(37.5)	
	>60 PY	6	(6.8)	2	(6.3)	4	(7.1)	
Alcohol drinking	Never	11	(12.5)	6	(18.8)	5	(8.9)	0.2740
	Former	26	(29.5)	7	(21.9)	19	(33.9)	
	Current	51	(58.0)	19	(59.4)	32	(57.1)	
Alcohol categories	0 g/day	16	(18.2)	7	(21.9)	9	(16.1)	**0.0140**
	1–30 g/day	39	(44.3)	20	(62.5)	19	(33.9)	
	31–60 g/day	11	(12.5)	2	(6.3)	9	(16.1)	
	>60 g/day	22	(25.0)	3	(9.4)	19	(33.9)	
Diagnostic outcome	HR-HPV DNA^+^ p16^+^	28	(31.8)	28	(87.5)	0	--	<0.0001
	HPV16 DNA^+^ RNA^+^	4	(4.5)	4	(12.5)	0	--	
	HR-HPV DNA^−^ p16^+^	8	(9.1)	0	--	8	(14.3)	
	HR-HPV DNA^−^ p16^−^	17	(19.3)	0	--	17	(30.4)	
	HPV16 DNA^+^ RNA^−^ p16^−^	25	(28.4)	0	--	25	(44.6)	
	HR-HPV DNA^−^	5	(5.7)	0	--	5	(8.9)	
	HPV16 DNA^+^ RNA^−^	1	(1.1)	0	--	1	(1.8)	
p16 Status	p16^+^	36	(40.9)	28	(87.5)	8	(14.3)	**<0.0001**
	p16^−^	42	(47.7)	0	--	42	(75.0)	
	*missing*	*10*	*(11.4)*	*4*	*(12.5)*	*6*	*(10.7)*	
TNM 2010 UICC	Stage I	10	(11.4)	3	(9.4)	7	(12.5)	0.6795
	Stage II	5	(5.7)	1	(3.1)	4	(7.1)	
	Stage III	18	(20.5)	7	(21.9)	11	(19.6)	
	Stage IVA	43	(48.9)	17	(53.1)	26	(46.4)	
	Stage IVB	11	(12.5)	3	(9.4)	8	(14.3)	
	Stage IVC	1	(1.1)	1	(3.1)	0	--	
TNM 2017 UICC	Stage I	18	(20.5)	9	(28.1)	9	(17.9)	**0.0008**
	Stage II	19	(21.6)	13	(40.6)	6	(10.7)	
	Stage III	15	(17.0)	4	(12.5)	11	(19.6)	
	Stage IVA	16	(18.2)	4	(12.5)	12	(21.4)	
	Stage IVB	19	(21.6)	1	(3.1)	18	(32.1)	
	Stage IV	1	(1.1)	1	(3.1)	0	(0.0)	
T categories TNM	T1	19	(21.6)	5	(15.6)	14	(25.0)	0.0349
2017	T2	29	(33.0)	13	(40.6)	16	(28.6)	
	T3	24	(27.3)	8	(25.0)	16	(28.6)	
	T4	4	(4.5)	4	(12.5)	0	--	
	T4a	10	(11.4)	1	(3.1)	9	(16.1)	
	T4b	2	(2.3)	1	(3.1)	1	(2.3)	
N categories TNM	N0	21	(23.9)	7	(21.9)	14	(25.0)	0.8349
2010	N1	14	(15.9)	6	(18.8)	8	(14.3)	
	N2a	3	(3.4)	1	(3.1)	2	(3.6)	
	N2b	22	(25.0)	10	(31.3)	12	(21.4)	
	N2c	19	(21.6)	6	(18.8)	13	(23.3)	
	N3	9	(10.2)	2	(6.3)	7	(12.5)	
N categories TNM	N0	21	(23.9)	7	(18.8)	14	(26.8)	**0.0006**
2017	N1	19	(21.6)	11	(34.4)	8	(14.3)	
	N2	14	(15.9)	10	(31.3)	4	(7.1)	
	N2a	5	(5.7)	1	(3.1)	4	(7.1)	
	N2b	2	(2.3)	1	(3.1)	1	(1.8)	
	N2c	7	(8.0)		--	7	(12.5)	
	N3	1	(2.3)	1	(3.1)	0	--	
	N3b	19	(21.6)	1	(3.1)	18	(32.1)	
Localization	Oropharynx ^3^	12	(13.6)	3	(9.4)	9	(16.1)	0.0915
	Tonsils ^4^	31	(35.2)	18	(56.3)	13	(23.2)	
	Base of tongue ^5^	10	(11.4)	3	(9.4)	7	(12.5)	
	Tongue ^6^	9	(10.2)	3 ^7^	(9.4)	6	(10.7)	
	Floor of mouth ^8^	5	(5.7)	1	(3.1)	4	(7.1)	
	Hypopharynx ^9^	7	(8.0)	2	(6.3)	5	(8.9)	
	Larynx ^10^	14	(15.9)	2	(6.3)	12	(21.4)	

^1^ HPV-driven: p16^+^ and HR-HPV DNA^+^ (*n* = 28) or HPV16DNA^+^RNA^+^ (*n* = 4); ^2^ not HPV-driven: other HNSCC; ^3^ ICD-10-C10; ^4^ ICD-10-C09; ^5^ ICD-10-C01; ^6^ ICD-10-C02; ^7^ including one ICD-10-C02 patient staged as p16^+^ OPSCC; ^8^ ICD-10-C04; ^9^ ICD-10-C12 and ICD-10-C13; ^10^ ICD-10-C32; * *p*-value according to *Pearson’s* chi-square (*χ*^2^) test, two-sided. Significant *p*-values < 0.05 are bold.

**Table 2:** Detection of HR-HPV DNA in HPV-driven and not HPV-driven oropharyngeal squamous cell carcinoma (OPSCC) and other head and neck squamous cell carcinomas (other) in pre- and post-therapy mouthwashes.

Mouth-wash	Localization	Finding/HPV Detection	Total *n* (%)	HPV-Driven *n* (%)	Not HPV-Driven *n* (%)	*p*-Value ^$^	*p*-Value ^§^
Pre	OPSCC ^†^	HR-HPV DNA-negative	21 (67.7)	5 (41.7)	16 (84.2)	**0.0136**	**0.0214**
		HR-HPV DNA-positive	10 (32.3)	7 (58.3)	3 (15.8)		
	Other ^‡^	HR-HPV DNA-negative	23 (82.1)	4 (66.7)	19 (86.4)	0.2641	0.2855
		HR-HPV DNA-positive	5 (17.9)	2 (33.3)	3 (13.6)		
	Total	HR-HPV DNA-negative	44 (74.6)	9 (50.0)	35 (85.4)	**0.0041**	**0.0078**
		HR-HPV DNA-positive	15 (25.4)	9 (50.0)	6 (14.6)		
Post	OPSCC ^†^	HR-HPV DNA-negative	41 (83.7)	21 (87.5)	20 (80.0)	0.4777	0.7019
		HR-HPV DNA-positive	8 (16.3)	3 (12.5)	5 (20.0)		
	Other ^‡^	HR-HPV DNA-negative	28 (96.6)	7 (100)	21 (95.5)	0.5659	>0.999
		HR-HPV DNA-positive	1 (3.4)	--	1 (4.5)		
	Total	HR-HPV DNA-negative	69 (88.5)	28 (90.3)	41 (87.2)	0.6761	>0.999
		HR-HPV DNA-positive	9 (11.5)	3 (9.7)	6 (12.8)		

^$^*p*-value according to *Pearson’s* chi-square (*χ*^2^) test, two-sided; ^§^ *p*-value according to *Fisher’s* exact test, two-sided; ^†^ localization of the primary lesion in the oropharynx (ICD-10-C01, C09, C10); ^‡^ primary head and neck squamous cell carcinoma outside the oropharynx (ICD-10-C02, C04, C12, C13, C32). Significant *p*-values < 0.05 are bold.

**Table 3:** Detection of HPV16 DNA in HPV-driven and not HPV-driven oropharyngeal squamous cell carcinoma (OPSCC) and other head and neck squamous cell carcinoma (other) in pre- and post-therapy mouthwashes.

Mouth-wash	Localization	Finding/HPV Detection	Total *n* (%)	HPV-Driven *n* (%)	Not HPV-Driven *n* (%)	*p*-Value ^$^	*p*-Value ^§^
pre	OPSCC ^†^	HPV16 DNA-negative	27 (87.1)	9 (75.0)	18 (94.7)	0.1103	0.2718
		HPV16 DNA-positive	4 (12.9)	3 (25.0)	1 (5.3)		
	Other ^‡^	HPV16 DNA-negative	25 (89.3)	4 (66.7)	21 (95.5)	**0.0433**	0.1068
		HPV16 DNA-positive	3 (10.7)	2 (33.3)	1 (4.5)		
	Total	HPV16 DNA-negative	52 (88.1)	13 (72.2)	39 (95.1)	**0.0123**	**0.0229**
		HPV16 DNA-positive	7 (11.9)	5 (27.8)	2 (4.9)		
post	OPSCC ^†^	HPV16 DNA-negative	47 (95.9)	24 (100)	23 (92.0)	0.1571	0.4898
		HPV16 DNA-positive	2 (4.1)	--	2 (8.0)		
	Other ^‡^	HPV16 DNA-negative	29 (100)	7 (100)	22 (100)	--	--
		HPV16 DNA-positive	--	--	--		
	Total	HPV16 DNA-negative	76 (97.4)	31 (100)	45 (95.7)	0.2446	0.5148
		HPV16 DNA-positive	2 (2.6)	--	2 (4.3)		

^$^ *p*-value according to *Pearson’s* chi-square (*χ*^2^) test, two-sided; ^§^ *p*-value according to *Fisher’s* exact test, two-sided; ^†^ localization of the primary lesion in the oropharynx (ICD-10-C01, C09, C10); ^‡^ primary head and neck squamous cell carcinoma outside the oropharynx (ICD-10-C02, C04, C12, C13, C32). Significant *p*-values < 0.05 are bold.

**Table 4:** Numbers of patients with HPV-driven and other head and neck squamous cell carcinoma (other), as well as recurrences according to detection of HR-HPV DNA in post-therapy mouthwashes.

HPV-Driven vs. Other HNSCC	Patient Groups		Case Number Per Group	Number of Events	Censored ^†^	
	Pre-Therapy Mouthwash	Post-Therapy Mouthwash	*n*	*n*	*n*	(%)
Not HPV-driven	Other		56	19	37	(66.1)
HPV-driven	HR-HPV detected	Loss of HR-HPV ^‡^	5	0	5	(100.0)
		Persistent HR-HPV	3	2	1	(33.3)
	Other		24	3	21	(87.5)
	Total		32	5	27	(84.4)
Total			88	24	64	(72.7)

^†^ Patients alive without any sign of disease at last follow-up visit; ^‡^ these five cases with HPV-driven OPSCC were all HPV16 DNA-positive without detection of other HR-HPV subtypes in pre-therapy mouthwashes and HR-HPV DNA-negative within mean follow-up of 22.3 (range 10.2–27.1) months.

## Data Availability

The data will be made available upon reasonable request.
